# Evaluation of optic nerve head blood flow in response to increase of intraocular pressure

**DOI:** 10.1038/s41598-018-35683-y

**Published:** 2018-11-22

**Authors:** Takeshi Iwase, Tomohiko Akahori, Kentaro Yamamoto, Eimei Ra, Hiroko Terasaki

**Affiliations:** Department of Ophthalmology, Nagoya University Graduate School of Medicine, Nagoya, Japan

## Abstract

The time course of the changes in the optic nerve head (ONH) blood flow in response to changes in the ocular perfusion pressure (OPP) induced by an artificial elevation of the intraocular pressure (IOP) has not been determined. We measured the blood flow, represented by the mean blur rate (MBR), on the ONH determined by laser speckle flowgraphy. The MBR was determined before, during, and after the IOP was elevated by 20 or 30 mmHg by pressure applied on the eye by an ophthalmodynamometer in a total of 27 healthy eyes. For an IOP elevation of 20 mmHg, the percentage reduction in the MBR-vessel was −24.7%, and in the MBR-tissue was −16.0% (*P* < 0.001). For an IOP elevation of 30 mmHg, the percentage reduction of the MBR-vessel was −35.3% and the MBR-tissue was −24.7% (*P* < 0.001). During the 30 mmHg IOP elevation for 10 minutes, both the MBR-vessel and MBR-tissue began returning to the baseline level from 1 minute after the beginning of the IOP elevation (*P* < 0.01, *P* < 0.05, respectively) and continued returning during the 10 minutes IOP elevation (*P* < 0.001, *P* < 0.01, respectively). We conclude that the ONH can autoregulate its blood flow in response to experimental changes in OPP induced by IOP elevations.

## Introduction

Autoregulation plays an important role in controlling the blood flow in tissues of the different organs in the body, and autoregulation enables a constant supply of oxygen and nutrients to the tissues to maintain the homeostasis^1^. Autoregulation is achieved by altering the resistance to blood flow by changing the blood vessel tone which involves dilatation or constriction of the vessels in response to decreased perfusion pressure (PP).

Autoregulation of the ocular blood flow in response to changes in the intraocular pressure (IOP) has been investigated by various methods and in different species^1,2^.

The results have shown that the blood supply to the retina and optic nerve head (ONH) is well autoregulated. The autoregulation of the blood flow on the ONH during changes in the ocular PP (OPP) by increases in the IOP with a constant mean ophthalmic artery pressure has been investigated^3–8^. However, the level of the OPP below which autoregulation in the ONH breaks down has been different in these different experimental studies. In addition, these earlier studies have mainly focused on whether autoregulation was present and its functional range. Most of these earlier studies evaluated the changes in the ONH blood flow at various time points after the changes in the OPP. However, the time course of the autoregulatory responses have not been fully investigated especially in humans.

Clinically, the IOP is elevated not only in glaucoma attack, but also under different situations. A transient increase in the IOP is a well-known complication of intravitreal injections^9–11^. Gismondi *et al*. reported that 88.9% of eyes (54 eyes in total) had an IOP of more than 30 mmHg for 5 seconds after an intravitreal injection^12^, whereas Kim *et al*. reported a mean IOP immediately postinjection was 44 mmHg (120 eyes in total) with 36% of eyes exceeding 50 mm Hg^13^. In addition, the IOP is intentionally elevated during LASIK surgery^14^ and during vitrectomy to block accidental hemorrhage from the retina or choroid. Even in other fields, e.g. urology surgery, the IOP increases with steep Trendelenburg positioning during robotic-assisted laparoscopy^15^. Therefore, it is important to know how the ONH blood flow responds to continuous IOP elevations.

A variety of techniques have been developed to measure the ocular blood flow. Scanning laser Doppler flowmetry (LDF)^4,16^ and color Doppler imaging can quantify the rate of ocular blood flow^17,18^, but it is difficult to record from the same site because the recording area is very small. One good approach to overcome these problems is the measurement of the blood flow velocities using laser Doppler velocimetry and measurement of the vessel diameters^8,19^. However, the clinical use of this technique is limited because it is time-intensive.

Because the ONH blood flow is well autoregulated, the time the blood flow is measured after the IOP elevation is important. Accordingly, measurements made at different time points could lead to different results.

A relatively new device to measure the ocular blood flow is the laser speckle flowgraphy (LSFG) instrument. It is a non-invasive, real-time method that has been used to measure the relative blood flow rates without the use of intravenous contrast agents^20–22^. The changes in the speckle pattern represent alterations in the relative blood flow rates in the retinal and ONH blood vessels, and the changes are represented by the mean blur rate (MBR). The MBR images are acquired at a rate of 30 frames/s over a 4-s period. The measurements have excellent reproducibility with a coefficient of variation (COV) of 4.7^23^. Therefore, LSFG is considered to be a practical and rapid method of evaluating the changes in the blood flow rate on the ONH caused by alterations of the OPP induced increases of the IOP.

The purpose of this study was to determine the autoregulation of the blood flow rate on the ONH of normal eyes. The time course of the changes in the ONH blood flow was determined by LSFG in response to changes in the OPP induced by an artificial elevation of the IOP.

## Results

**Figure 1:**
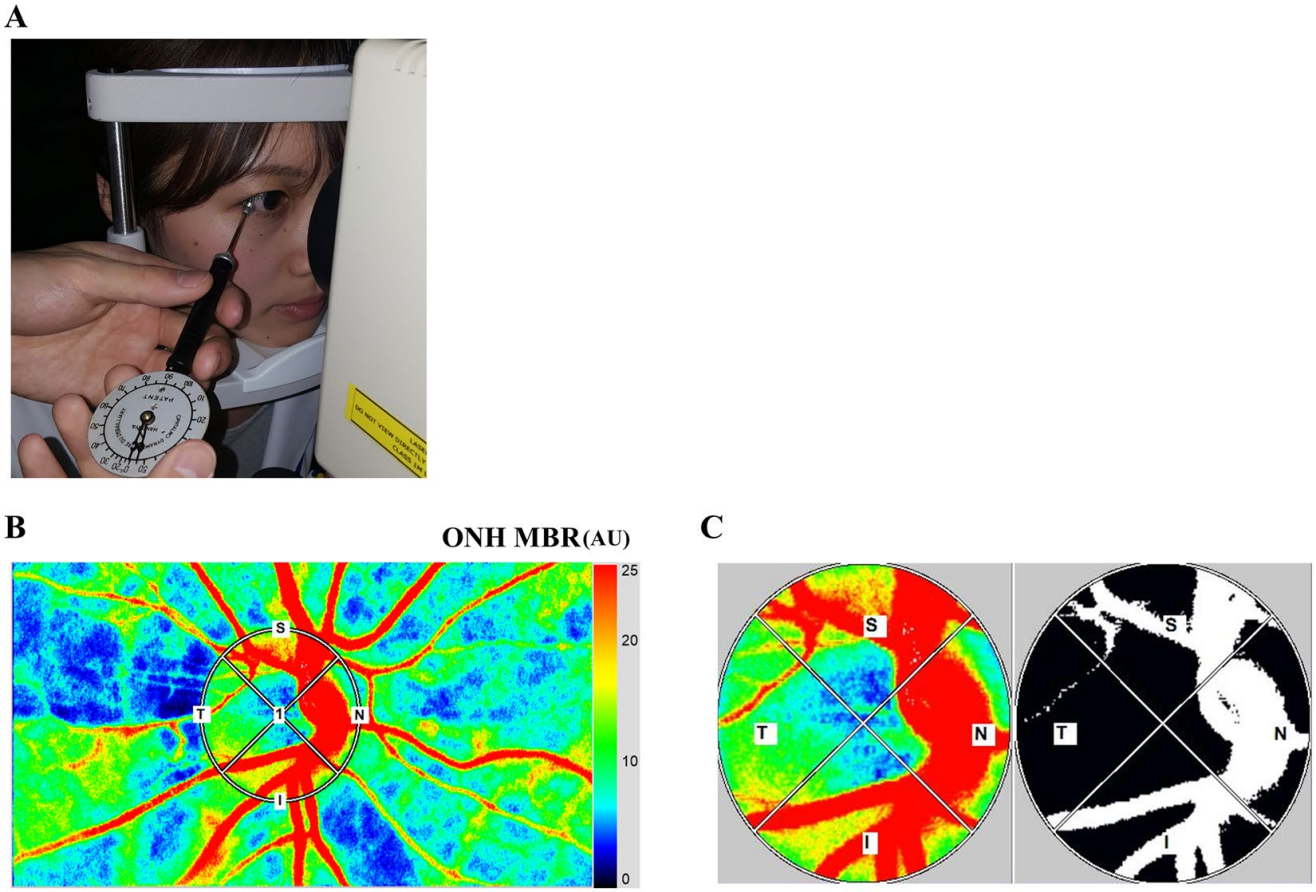
Photographs of images acquired by laser speckle flowgraphy (LSFG) with the ophthalmodynamometer in place (**A**). To measure the MBR of the blood flow on the optic nerve head (ONH) a circle was set around the ONH. Red color indicates a high MBR and the blue color indicates a low MBR (**B**). Binary image for segmentation between the vessel (white area) and tissue (black area) areas (**C**).

**Figure 2:**
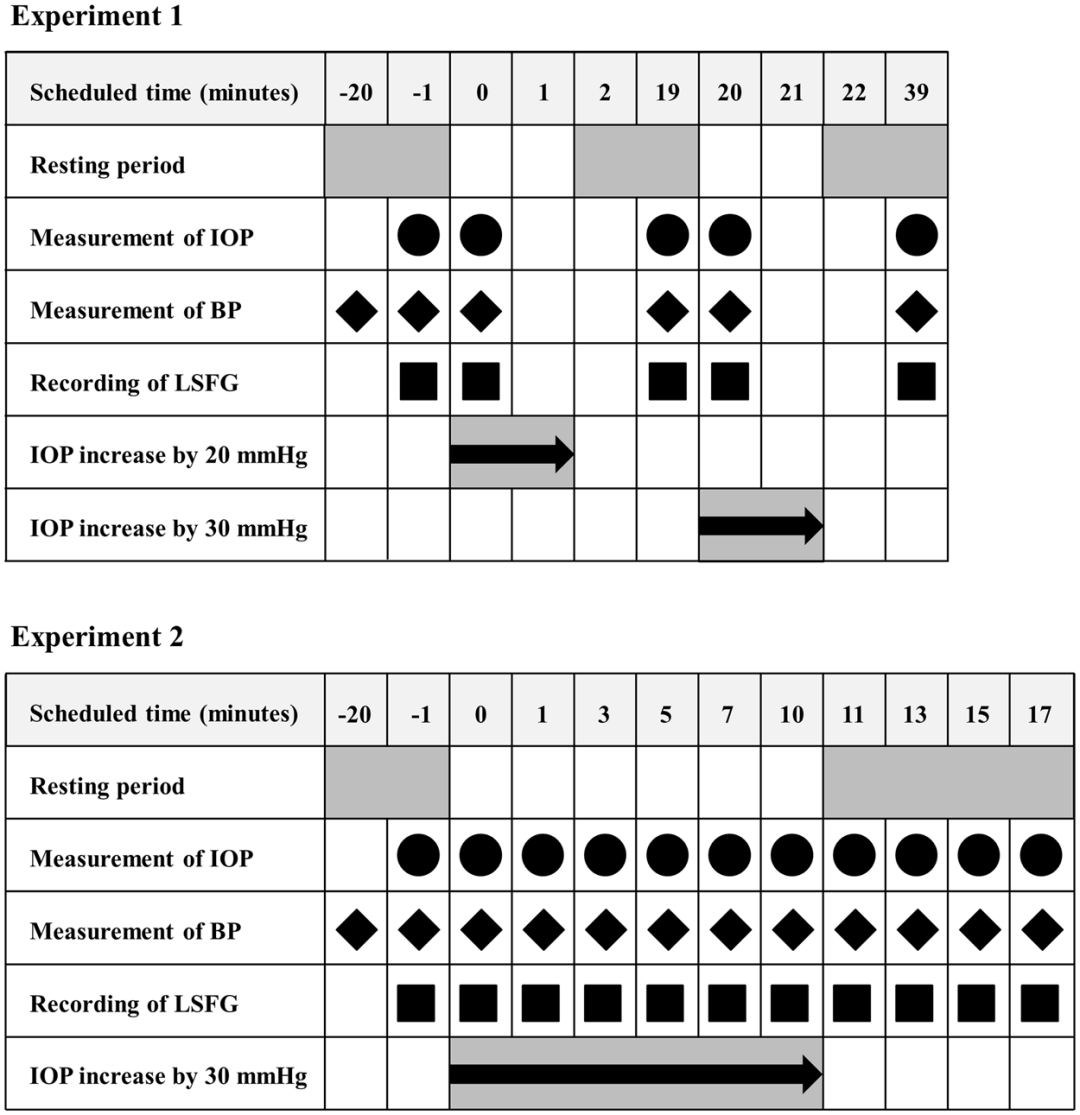
Illustration of the time course of the experiments.

**Figure 3:**
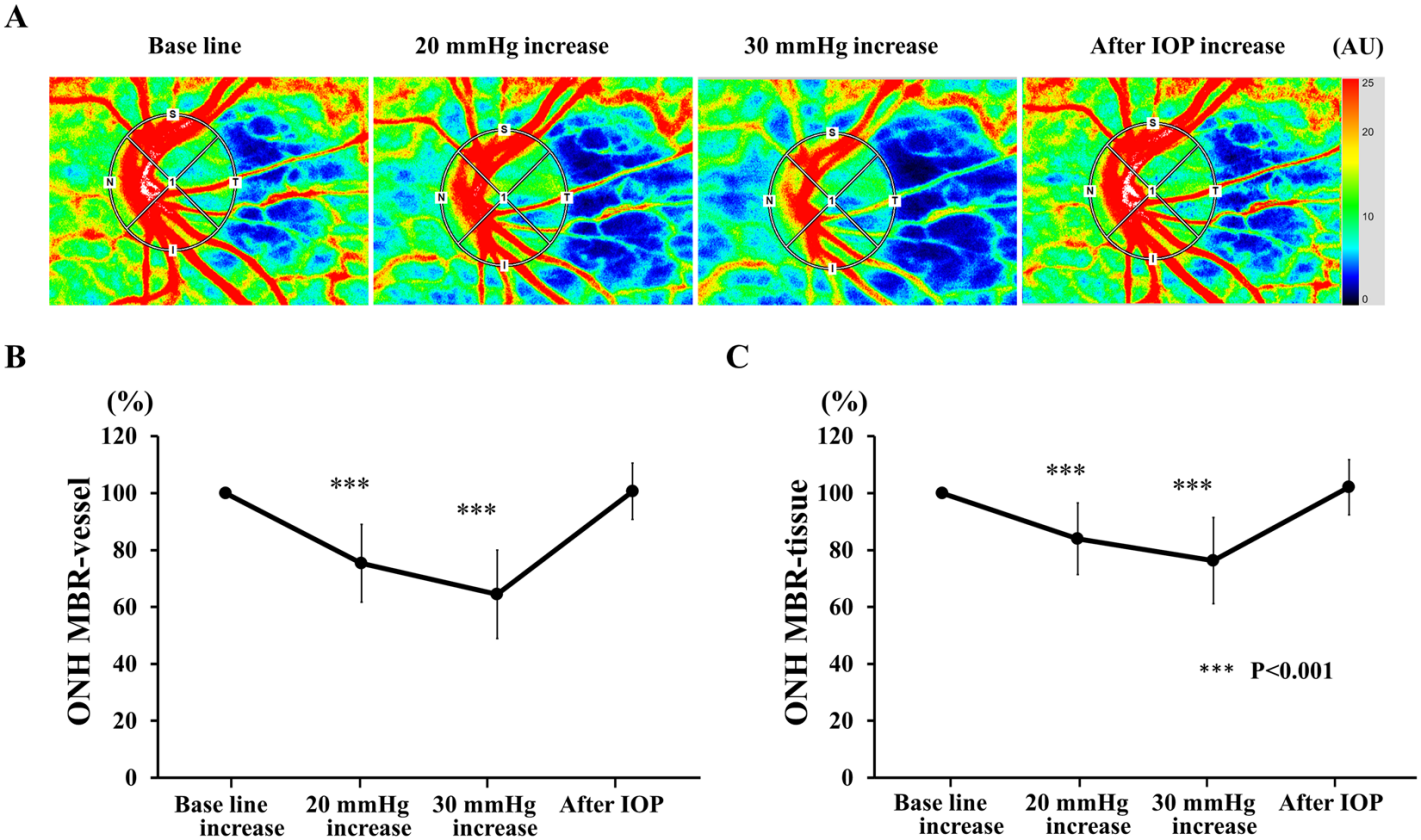
Representative composite colour maps using the mean blur rate (MBR) as measured by LSFG in a healthy eye before the IOP elevation, during 20 and 30 mmHg IOP elevation, and after the IOP elevation (**A**). There was a significant reduction in the optic nerve head (ONH) MBR-vessel (**B**) and MBR-tissue (**C**) during the IOP increase. ***P < 0.001

In Experiment 1, the IOP was increased by 20 or 30 mmHg from the baseline for 1 minute (Fig. 1). The LSFG images were recorded at 1 minute before the IOP elevation, immediately after the IOP elevations, and at 20 minutes after the release of the pressure (Figs 2 and 3). Experiment 2 was conducted on a completely different set of subjects, and the IOP was increased by 30 mmHg from the baseline for 10 minutes. The LSFG images were recorded before, immediately after the IOP elevation, time 0, and at 1 min (time 1), 3 min (time 3), 5 min (time 5), 7 min (time 7), and 10 min (time 10) while the IOP was elevated. The LSFG images were also recorded at 1 min (time 11), 3 min (time 13), and 5 min (time 15) after the release of the pressure on the eye (Fig. 2).

### Subject dispositions, demographics, and baseline characteristics

Thirty healthy Japanese individuals were recruited for the experiments, and two subject dropped out immediately after the application of the ophthalmodynamometer on the conjunctiva though they could not tolerate the pain in the Experiment 1, and one subject was excluded from Experiment 1 because his systolic blood pressure (SBP) was >150 mmHg. In the end, 27 subjects were studied in this study.

Seventeen volunteers with an average age of 32.7 ± 7.9 years completed all phases of the examinations in Experiment 1, and 10 volunteers with an average age of 33.2 ± 8.3 years completed all phases of Experiment 2. The demographics of the volunteers for Experiment 1 are shown in Table 1 and that for the volunteers for Experiment 2 are shown in Table 2. No adverse events were observed in any of the volunteers during or after the measurements.

**Table 1:** Baseline Characteristics of Subjects (Experiment 1).

Characteristics (n = 17)	mean ± SD
Age (years)	32.7 ± 7.9
IOP (mmHg)	15.7 ± 3.9
Axial length (mm)	25.7 ± 0.94
Refractive error (diopter)	−4.12 ± 2.59
Systolic blood pressure (mmHg)	116.7 ± 13.2
Diastolic blood pressure (mmHg)	72.2 ± 10.1
Heart rate (bpm)	69.4 ± 6.5

**Table 2:** Baseline Characteristics of Subjects (Experiment 2).

Characteristics (n = 10)	mean ± SD
Age (years)	33.2 ± 8.3
IOP (mmHg)	15.7 ± 3.6
Axial length (mm)	25.8 ± 0.94
Refraction (diopter)	−4.26 ± 2.54
Systolic blood pressure (mmHg)	116.9 ± 13.8
Diastolic blood pressure (mmHg)	72.3 ± 10.6
Heart rate (BPM)	69.7 ± 6.8

### Changes in intraocular pressure (IOP) and ocular perfusion pressure (OPP)

A significant increase in the IOP was observed after applying the ophthalmodynamometer in both experiments but no significant changes in the systolic blood pressure (SBP), diastolic blood pressure (DBP), and mean arterial blood pressure (MAP) were observed. The results showed that the application of the ophthalmodynamometer caused a significant increase in the IOP of 20 mmHg (+141.8%), and 30 mmHg (+209.1%). The MAP was not significantly changed under both pressures (Table 3). The increase in the IOP by 20 mmHg reduced the OPP by 50.9% and the increase by 30 mmHg reduced the OPP by 74.2% (both *P* < 0.001).

**Table 3:** Changes in parameters with IOP increase.

	Base line	20 mmHg IOP increase	30 mmHg IOP increase	After release of the pressure	*P*-value
MAP (mmHg) (%)	87.0 ± 10.8 100	86.7 ± 9.2 99.6 ± 5.6	86.3 ± 9.2 99.2 ± 8.2	85.1 ± 7.7 97.8 ± 7.6	0.943
IOP (mmHg) (%)	15.7 ± 3.9 100	36.5 ± 5.1 241.8 ± 49.2	46.0 ± 3.7 309.1 ± 77.2	15.2 ± 3.2 96.8 ± 10.6	<0.001
OPP (mmHg) (%)	42.3 ± 8.4 100	21.3 ± 8.4 49.1 ± 12.2	11.5 ± 7.6 25.8 ± 17.0	41.5 ± 5.7 99.5 ± 4.7	<0.001
MBR-vessel (AU) (%)	46.8 ± 4.7 100	35.4 ± 8.0 75.3 ± 13.6	30.5 ± 8.9 64.7 ± 16.9	47.2 ± 6.8 100.1 ± 9.9	<0.001
MBR-vessel (AU) (%)	11.9 ± 1.8 100	10.0 ± 2.3 84.0 ± 12.6	9.0 ± 2.1 75.3 ± 18.0	12.2 ± 2.7 102.1 ± 9.7	<0.001

**Figure 4:**
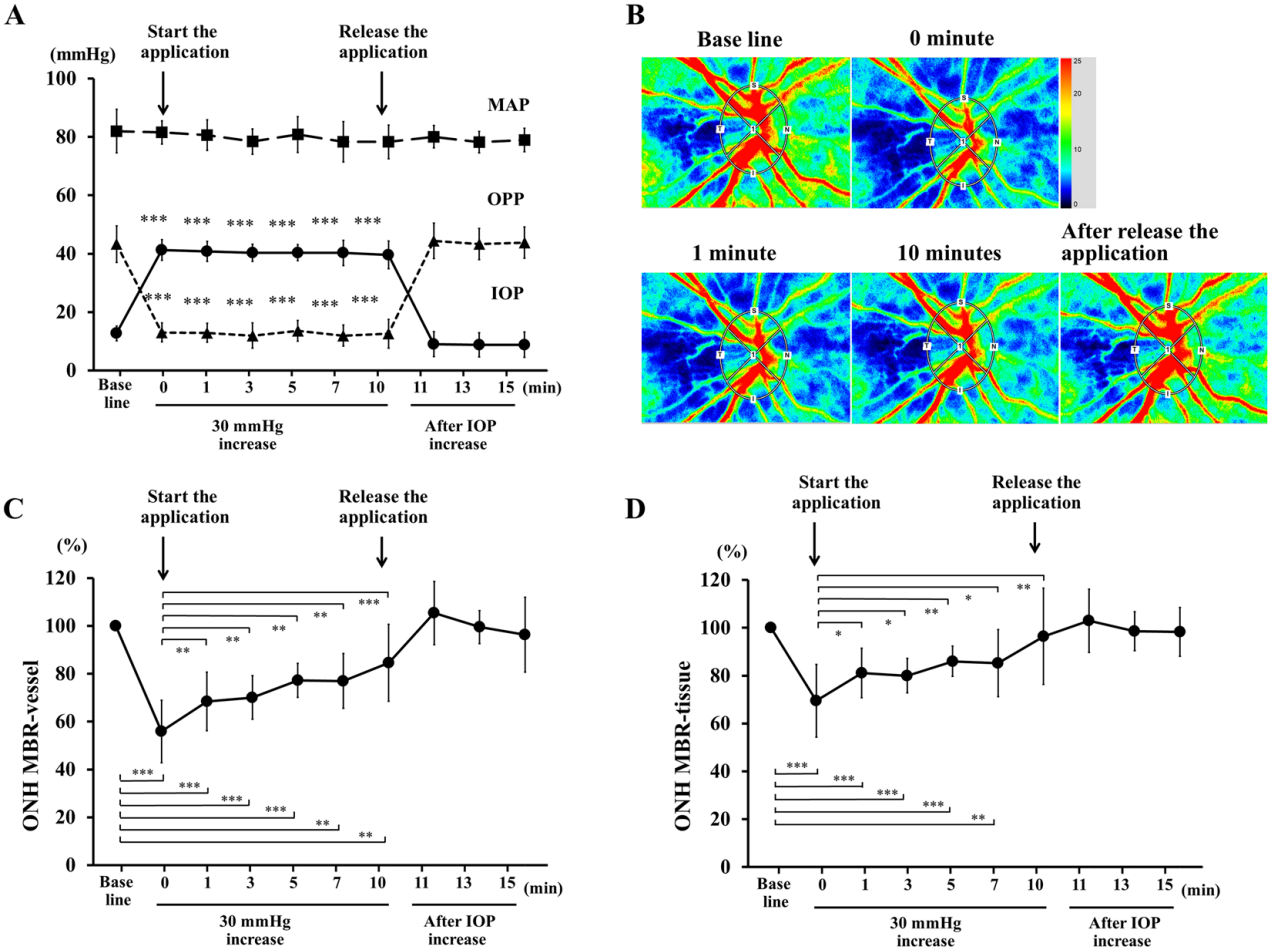
Changes in the systemic and ocular parameters before an increase in the IOP, during 30 mmHg IOP increases for 10 minutes, and after the IOP was returned to baseline pressure. The IOP was significantly increased, and the OPP was significantly decreased during the application of the ophthalmodynamometer but the MAP was not changed (**A**). Representative composite colour maps using the MBR as measured by LSFG (**B**). MBR-vessel was significantly reduced from immediately after the IOP elevation by 30 mmHg until the pressure was released (**C**). During the 30 mmHg IOP elevation, the MBR-vessel significantly recovered from 1 minute through 10 minutes during 30 mmHg IOP elevation compared to that immediately after IOL elevation. The MBR-tissue was significantly reduced from immediately to 7 minutes during the IOP elevation by 30 mmHg but it was not significantly reduced 10 minutes after the IOP elevation (**D**). During the 30 mmHg IOP elevation, the MBR-tissue significantly recovered from 1 minute through 10 minutes during 30 mmHg IOP elevation compared to that immediately after IOL elevation. ***P < 0.001, **P < 0.01, *P < 0.05

In Experiment 2, the application of the ophthalmodynamometer caused a stable and significant increase in the IOP by 30 mmHg for 10 minutes, and the MAP did not change significantly (Fig. 4A). Thus, the OPP was significantly decreased during the IOP elevation. After the release of the pressure, the IOP and OPP returned close to the baseline pressure.

### Changes in mean blur rate (MBR) of ONH in Experiment 1

In Experiment 1, the mean MBR-vessel was 46.8 ± 4.7 arbitrary units (AU) before, 35.4 ± 8.0 AU (−24.7%) during the 20 mmHg elevation and 30.5 ± 8.9 (−35.3%) AU during the 30 mmHg elevation. The MBR returned to 47.2 ± 6.8 AU at 20 min after the IOP was returned to the baseline IOP.

The mean MBR-tissue was 11.9 ± 1.8 arbitrary units (AU) before, 10.0 ± 2.3 AU (−16.0%) during the 20 mmHg elevation, and 9.0 ± 2.1 (−24.7%) AU during the 30 mmHg elevation. The MBR returned to 12.2 ± 2.7 AU at 20 min after the IOP elevation. The reductions in the MBR-vessel and MBR-tissue were significant during both levels of IOP elevations compared to the baseline values (both *P* < 0.001). The reductions of the ONH MBR-vessel and MBR-tissue were significantly less than that of the OPP during the 20 mmHg (both *P* < 0.001) and 30 mmHg (both *P* < 0.001).

### Changes in mean blur rate (MBR) of ONH in Experiment 2

In Experiment 2, the MBR-vessel was 39.4 ± 5.8 AU before the IOP elevation, and it was significantly reduced to 22.5 ± 7.6 AU (−42.8%) immediately after the IOP was elevated by 30 mmHg (Fig. 4B). The MBR-vessel remained significantly reduced until the pressure was released (time 0 to time 5, *P* < 0.001, time 7 to time 10, *P* < 0.01; Fig. 4C). During the 30 mmHg IOP elevation, the MBR-vessel significantly increased from time 0 (22.5 ± 7.6 AU, −42.8%) to time 1 (26.9 ± 6.9 AU, −31.6%; *P* < 0.01), and through time 10 (32.7 ± 7.3 AU, −17.1%; *P* < 0.001). After the release of the pressure, the MBR-vessel returned to 39.5 ± 2.5 AU (+5.4%) at time 11 to 39.2 ± 3.8 AU (−0.5%) at time 13, and 37.9 ± 5.1 AU (−3.6%) at time 15. All of these post-released values were not significantly different from that at the baseline.

The MBR-tissue was 10.4 ± 1.9 AU before the IOP elevation, and it was significantly reduced to 7.1 ± 2.5 AU (−32.0%) immediately after the IOP elevation by 30 mmHg. The MBR-tissue remained significantly reduced until 7 min after beginning the IOP elevation (time 0 to time 5, *P* < 0.001; time 7, *P* = 0.006; Fig. 4D), but the MBR increased and it was not significantly reduced at time 10 min. During the 30 mmHg IOP elevation, the MBR-tissue significantly increased from time 0 (7.1 ± 2.5 AU, −32.0%) to time 1 (8.4 ± 1.8 AU, −18.9%; *P* < 0.05) and through time 10 (9.7 ± 1.6 AU, −6.7%; *P* < 0.01). After the release of the pressure, the MBR-tissue returned to 10.4 ± 2.2 AU (+2.9%) at time 11, 10.2 ± 2.6 AU (−1.5%) at time 13, and 10.1 ± 1.6 AU (−2.2%) at time 15. All of these post-released values were not significantly different from that at the baseline.

### Correlation between MBR and other parameters

**Figure 5:**
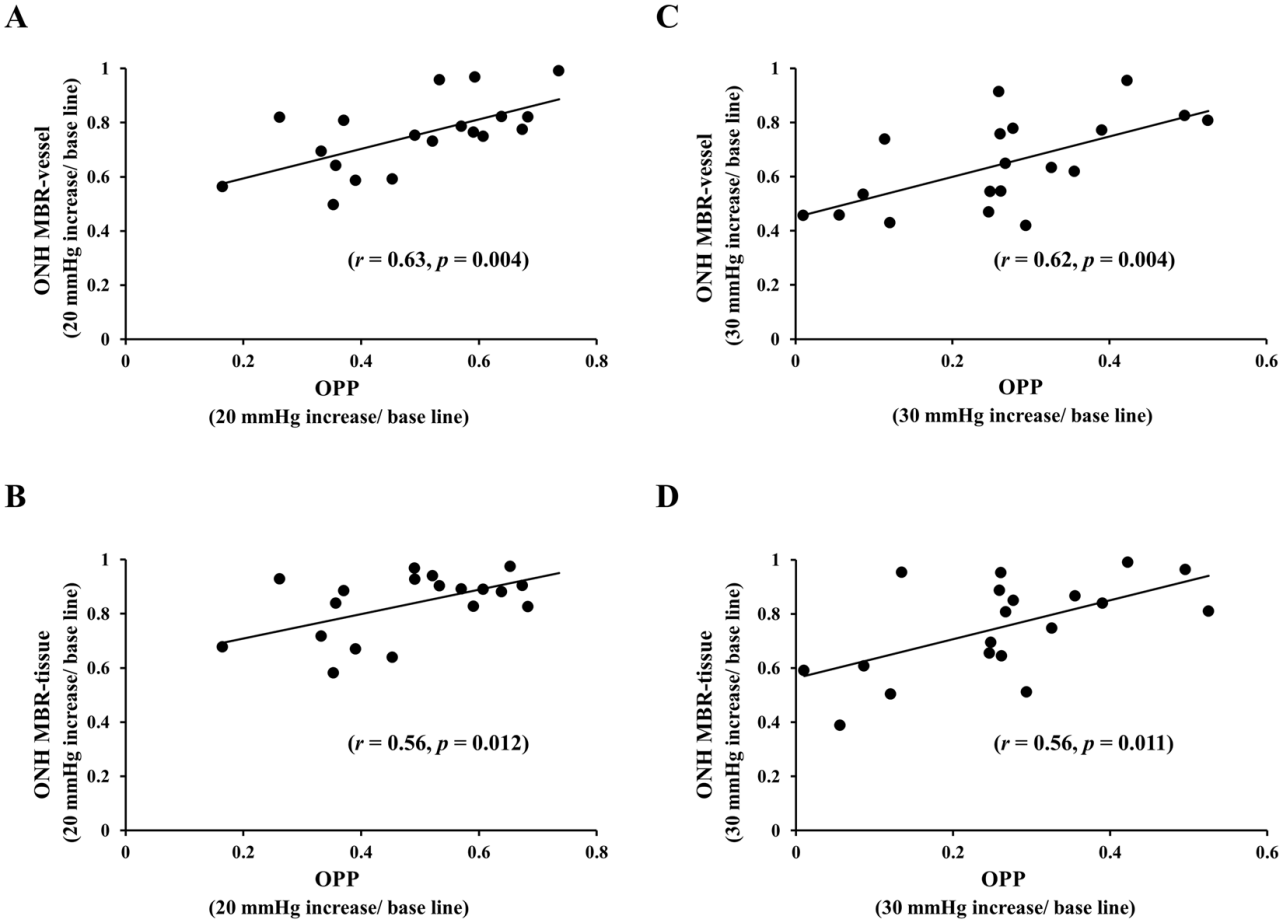
Relationship between the percentage reduction of the OPP and the MBR- vessel and MBR-tissue after the IOP elevation in the Experiment 1. The percentage reduction of the MBR-vessel was positively correlated with that of the OPP during the elevation of the IOP by 20 mmHg (*r* = 0.63, *P* = 0.004) (**A**) and by 30 mmHg (*r* = 0.62, *P* = 0.004) (**B**). The percentage reduction of the MBR -tissue was positively correlated with that of the OPP during the elevation of the IOP by 20 (*r* = 0.56, *P* = 0.012) (**C**) or 30 mmHg (*r* = 0.56, *P* = 0.011) (**D**).

In Experiment 1, plots of the percentage reduction of the OPP against the MBR-vessel and MBR-tissue during the IOP elevation are shown in Fig. 5. The percentage reduction of the MBR-vessel was positively and significantly correlated with the OPP during the elevation of the IOP by 20 mmHg (*r* = 0.63, *P* = 0.004) and by 30 mmHg (*r* = 0.62, *P* = 0.004). Also, the percentage reduction of the MBR-tissue was positively correlated with the OPP during the elevation of the IOP by 20 mmHg (*r* = 0.56, *P* = 0.012) and 30 mmHg (*r* = 0.56, *P* = 0.011).

**Figure 6:**
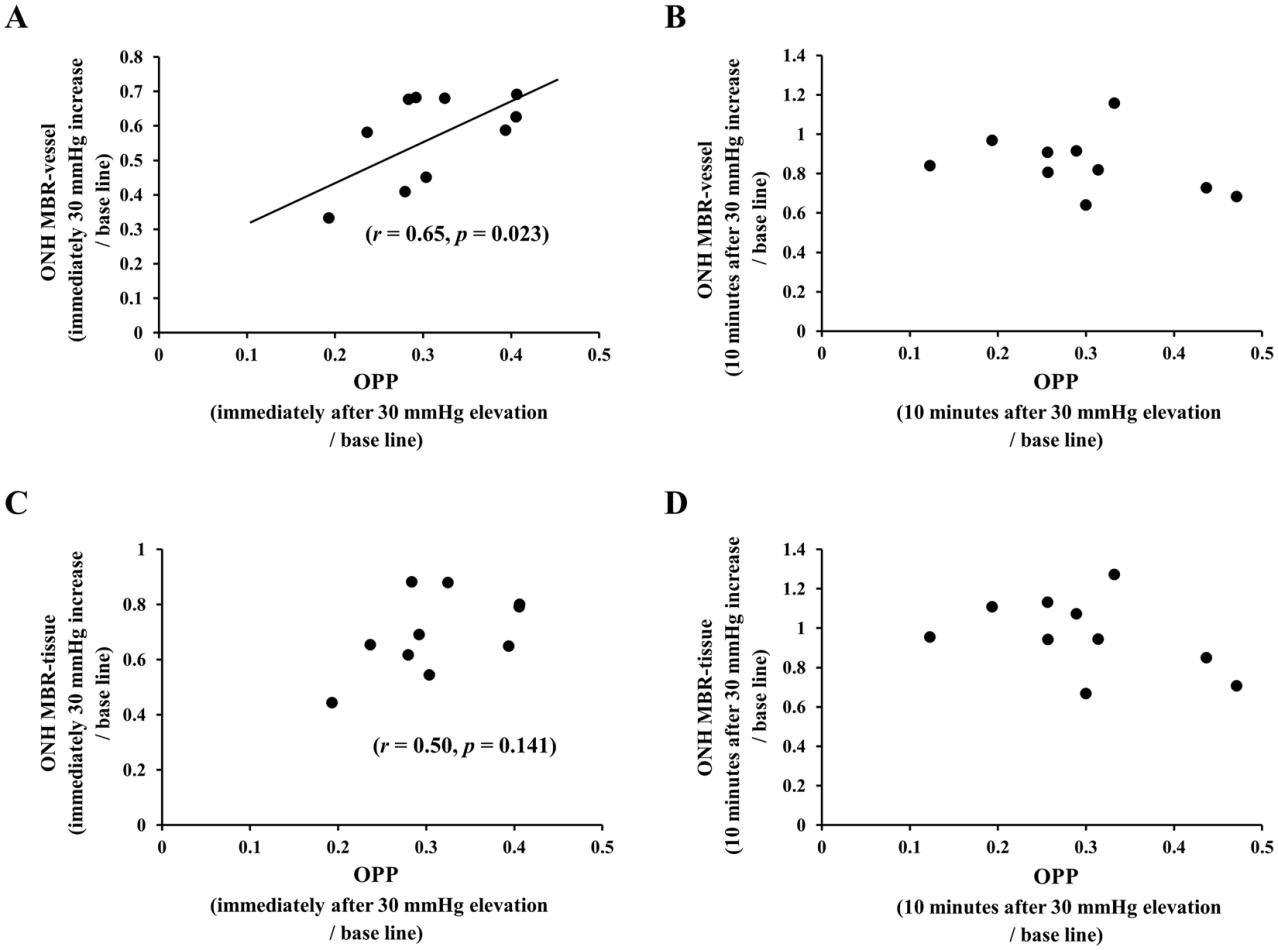
Correlation coefficients between the percentage reduction of the ONH MBR-vessel and OPP was 0.65 immediately after the 30 mmHg IOP elevation (*P* = 0.023) (**A**), but was reduced to 0.13 at 10 minutes during the IOP elevation (*P* = 0.758) (**B**). The correlation coefficient between the percentage reduction of the ONH MBR-tissue and OPP was 0.50 immediately after the 30 mmHg IOP elevation (*P* = 0.141) (**C**) but was reduced to 0.12 at 10 minutes during the IOP elevation (*P* = 0.780) (**D**).

In Experiment 2, the coefficients of correlation between the percentage reduction of the MBR-vessel (*r* = 0.65, *P* = 0.023) and MBR-tissue (*r* = 0.50, *P* = 0.141) and the OPP were similar to that in Experiment 1 at time 0. However, it was reduced at time 10 (Fig. 6).

## Discussion

Our results showed that the MBR-vessel of the ONH, corresponding to the retinal blood flow, MBR-tissue, corresponding to the capillary network of the ONH, and the OPP were significantly reduced immediately after the IOP elevation. All of the correlations between the reduction of the OPP and the ONH MBR-vessel and MBR-tissue were significant during both the 20 mmHg and 30 mmHg IOP elevation. During the 10 minutes of IOP elevation by 30 mmHg, both the ONH MBR-vessel and MBR-tissue began to return to the baseline values from 1 minute after the beginning of the IOP elevation. Because the MAP was not altered by the IOP elevation, the reduction in the ONH MBR must have been due to the decrease in the OPP. Thus, the IOP elevation appears to be the major factor causing the decrease in the ONH MBR.

Clinically, the IOP is elevated in different situations. Recently, intravitreal injections of anti-VEGF agents are commonly used for several retinal diseases, e.g. retinal vein occlusion, age-related macular degeneration, and others, and a transient increase in IOP is a well-known complication of intravitreal injections of anti-VEGF agents^9–11^. The IOP is elevated even with steep Trendelenburg positioning during robotic-assisted laparoscopy^15^. Therefore, it is important to know how the ONH blood flow responds to continuous IOP elevations.

In normal monkeys, autoregulation in the ONH has been reported to be active at OPPs >30 mmHg^24,25^, but autoregulation was not present at <30 mmHg^26^. In another study, a rapid increase of the IOP from 20 mmHg to either 40 or 50 mmHg induced no significant changes in the blood flow on the ONH of rabbits^27^, indicating an efficient autoregulation at these IOP elevations. Thus, autoregulation operates only within a certain critical range of OPPs, and it is not active when the OPP is below or above this critical range^1,28^.

In humans, the blood vessels on the ONH have some autoregulatory ability during experimental increases in the IOP^3–8^. Thus, the ONH blood flow did not decrease significantly until the OPP values were 30% below the baseline value^4,8^. Previous studies on the blood flow on the ONH showed that the blood flow remained constant until the IOP values reached 40 to 45 mmHg^3,4^. In our experiments, the OPP was reduced by 50.9% (21.3 mmHg) of the baseline value, and the IOP was increased by 141.8% (36.5 mmHg) after the IOP elevation of 20 mmHg. However, the ONH MBR-vessel (−24.7%) and MBR-tissue (−16.0%) were significantly reduced. It has been reported that there is an autoregulatory plateau, and after the lower limit of autoregulation level is exceeded, there is a linear decrease in the blood flow thereafter^4,29^. The OPP was reduced by 74.2% (11.5 mmHg) of the baseline value, and the IOP was increased by 209.1% (46.0 mmHg) after the IOP elevation of 30 mmHg. The ONH MBR-vessel (−35.3%) and MBR-tissue (−24.7%) were significantly more reduced than that after the IOP was elevated by 20 mmHg. These findings indicate that the autoregulatory range on the ONH could be exceeded even by a 20 mmHg IOP elevation after which there is a linear decrease in the MBR.

Our findings showed that the ratio of the reduction of the ONH MBR-vessel (−24.7%, −35.3%) or MBR-tissue (−16.0%, −24.7%) was significantly smaller than that of the OPP (−50.9%, −74.2%, respectively). These findings indicate that some degree of ONH blood flow regulation occurred immediately after an increase in the IOP because the decrease in the OPP was more than the decrease in the ONH MBR-vessel or MBR-tissue. These results corroborate some earlier studies in humans which reported that a decrease in the OPP was accompanied by a decrease in the ONH blood flow although it was proportionately less than the decrease in OPP^8^. This indicated that there is some degree of autoregulation of the ONH blood flow in response to a decrease in the OPP^8,30–33^.

Both the ONH MBR-vessel and MBR-tissue decreased immediately after an acute IOP elevation and recovered significantly after 1 minute of the continuous 30 mmHg IOP elevation. In addition, the coefficients of correlation between the percentage reduction of the MBR-vessel and MBR-tissue and the OPP was significant immediately after the 30 mmHg IOP elevation. However, it was reduced at 10 minutes during the IOP elevation. This confirmed previous findings that the ONH shows some degree of autoregulation during an experimental increase in the IOP^27,34^. Takayama *et al*. reported a short-term recovery in the ONH blood flow just after an increase in the IOP in rabbits^27^. In cats, the ONH blood flow recovered within 1 minute after the change in the OPP^34^. These results indicated that the ONH blood flow is strongly autoregulated.

In humans, there have been a few studies which found changes in the ONH blood flow during a continuous IOP increase. Hashimoto *et al*. reported that MBR-vessel and MBR-tissue were decreased 5 minutes after an IOP elevation but it partially recovered 10 minutes after beginning the IOP elevation in normal control human eyes^35,36^. In addition, Kiyota *et al*. reported that time course of changes in the ONH blood flow after artificial IOP elevation and the MBR-tissue on the ONH decreased significantly only after 20 mmHg IOP elevation (−15 to −20%) in normal control human eyes^37^. Our results corroborate these results. However, they measured the blood flow at only 5 and 10 minutes during the IOP elevation^35,36^, and details of the changes in the blood flow during the 10 min of IOP elevation were not presented.

One MBR image can be acquired in 4 seconds by the LSFG-NAVI device, and thus the MBR can be determined many times to follow the changes in the MBR. Interestingly, the ONH MBR-vessel and MBR-tissue significantly recovered from 1 minute after the IOP elevation, continued to decrease toward the baseline level during the 10 min of IOP elevation. This resulted in no significant difference in the ONH MBR-tissue between the baseline and 10 min after the IOP elevation. Although the ONH blood flow did largely recover because of autoregulation, it did not fully return to baseline levels. Our study was limited to 10 min of measurements for ethical reasons. Measurements for longer episodes of increased IOP are needed to determine whether the ONH blood flow recovers to the baseline levels.

Our results showed clearly that the measuring time points after the IOP elevation were very important for evaluating the blood flow on the ONH because of the autoregulation. The level of IOP above which the autoregulation breaks down has been reported to vary in experimental studies even in normal human subjects^3^.

Differences in the measurement techniques can cause the differences. Our results showed that differences in the time the measurements can also cause different results. With many of the measurement techniques, the time intensiveness has limited their ability to make multiple measurements in a short time.

The vasculature of the ONH is not under neural control and, as such, it is dominated by myogenic and metabolic mechanisms when the OPP is changed^29^. The myogenic responses are activated when the intraluminal pressure is changed and results in pressure-dependent membrane depolarizations associated with changes in the diameter of the blood vessel lumens. If some autoregulation is active on the ONH when the OPP is acutely decreased, the myogenic responses are probably activated. The myogenic responses would be related to the degree of ONH blood flow regulation immediately after an increase of IOP as in our experiments. When the IOP is elevated for a longer period of time, the resulting hypoxia could lead to the initiation of metabolic mechanisms releasing NO^2^. In the retina of cats, hypoxia induced a significant vasodilatation of retinal vessels and a significant increase in retinal blood flow. This was diminished when a NOS inhibitor was administered intravitreously^38^. Whether NO-synthase inhibition would affect ONH blood flow regulation during a longer period of IOP elevation cannot be investigated in humans for ethical reasons. However, it is likely that some metabolic mechanism e.g., NO regulation, would be activated during a longer period of IOP elevation.

The reduction of the ONH MBR-tissue was less than that of MBR-vessel throughout our experimental times. The MBR-vessel mainly corresponds to the retinal blood flow and the MBR-tissue corresponds mainly to the capillary network of the ONH. These results indicate that there was a significant difference between the regulatory behavior of the capillary network on the ONH and retinal vessels when the IOP was increased experimentally. Our results suggest that the capillary network on the ONH is more autoregulated than the retinal blood flow. The post-laminar ONH is nourished by branches of the posterior ciliary arteries either directly or through the circle of Zinn-Haller. These structures could regulate the blood flow when the blood flow is reduced by the increase in the IOP.

The ONH lacks a normal blood-brain barrier, providing circulating molecules such as endothelin and angiotensin direct access to the smooth muscle cells^39,40^. Therefore, we investigated the autoregulation for IOP elevation using normal subjects, but the autoregulation should be different in patients with glaucoma and other diseases. It has been reported that ONH blood flow in glaucoma patients was decreased more rapidly after artificial IOP elevation than that in normal subjects^41^, indicating that autoregulation is different between glaucoma patients and normal subjects. In addition, the autoregulation for IOP elevation in subjects with vascular dysregulation is different from normal subjects^39,40^.

This study has several limitations. First, the IOP was elevated by only 20 and 30 mmHg by the pressure applied by an ophthalmodynamometer. There have been many reports on the effects of stepwise elevations of the IOP by the suction cup method^8,30–33,42^. Because we investigated the changes in the blood flow after an elevation of the IOP in one step, it is not easy to compare our findings to those obtained by an elevation in a stepwise way. Second, our study was performed on relatively young subjects, and the results cannot be extrapolated to elderly subjects. Third, the duration of the IOP elevation was only 10 min because times longer than 10 min were painful. Fourth, we did not evaluate what kind of autoregulatory response was functioning when the IOP was elevated. However, measurements of vessel diameter on the ONH might determine whether the myogenic response was activated. Fifth, many subjects were myopic. The results may be different from that in non-myopic subjects because of the differences in the morphological features of the optic disc^43^. Further studies with a wider range of ages and a larger number of subjects who are not myopic and IOP elevation in a step-by-step manner are needed.

In conclusion, the blood flow on the ONH was immediately reduced by an acute elevation of the IOP, but the blood flow began to return to the baseline level from 1 minute after the elevation. The results indicate that the ONH has ability to autoregulate its blood flow rapidly in response to experimental changes in the OPP induced by an elevation of the IOP in normal subjects.

## Methods

### Ethics statement

The procedures used were approved by the Ethics Committee of the Nagoya University Hospital and registered with the University Hospital Medical Network (UMIN)-clinical trials registry (UMIN000024980). The study was conducted at the Nagoya University Hospital. The procedures conformed to the tenets of the Declaration of Helsinki, and an informed consent was obtained from all subjects after an explanation of the procedures to be used and the possible complications. In addition, a written informed consent was obtained to publish the findings and identifying images in future publications.

### Experimental protocol

All volunteers were asked to abstain from alcoholic and caffeinated beverages on the morning the examination. Only the right eye was used for all of the experiments. The pupil was dilated 30 minutes before the examinations, and the subjects rested in a quiet dark room for 10 to 15 min before the measurements to achieve stable hemodynamics conditions. All examinations were performed in the sitting position at approximately 12:00 h to avoid diurnal variations^22,44^. The axial lengths were measured by partial optical coherence interferometry (IOLMaster; Carl Zeiss Meditec, La Jolla, CA), and the IOP was measured with a handheld tonometer (Icare; TiolatOy, Helsinki, Finland). The SBP and DBP were measured with an automatic sphygmomanometer (CH-483C; Citizen, Tokyo, Japan). The MAP and OPP were calculated as:

$$\begin{array}{rcl}{\rm{MAP}} & = & {\rm{DBP}}+1/3({\rm{SBP}}-{\rm{DBP}});\\ {\rm{OPP}} & = & 2/3{\rm{MAP}}\mbox{--}{\rm{IOP}}.\end{array}$$

Eyes were excluded if the best-corrected visual acuity was <20/20, glaucoma was present, a history of ophthalmic or systemic disorders, previous ocular laser or incisional surgery in the experimental eye, SBP >150 mmHg, DBP >90 mmHg, axial length >27.0 mm, and medical conditions that could influence the hemodynamics of the eye such as diabetes, hypertension, arrhythmia, and vascular diseases.

### Experimental elevation of IOP

An ophthalmodynamometer (Inami, Tokyo, Japan) was used to apply a fixed external pressure on the eye through the lower eyelid with the device held perpendicular to the globe (Fig. 1A). The ophthalmodynamometer scale showed the force applied to the eye to increase the IOP. We permitted the subjects to close their eyes during the time when the measurements were not performed and to blink during the application of ophthalmodynamometer to minimize the discomfort. The IOP was measured during the application of the pressure with the Icare tonometer (Icare^®^; Tiolat Oy, Helsinki, Finland) before, during, and after the application of the pressure.

To measure the blood flow, one experimenter kept the ophthalmodynamometer in place and another experimenter recorded the LSFG images. One example of the time course of the changes in the IOP is shown in Fig. 2.

In Experiment 1, the IOP was increased by 20 or 30 mmHg from the baseline for 1 minute. The LSFG images were recorded at 1 minute before the IOP elevation, immediately after the IOP elevations, and at 20 minutes after the release of the pressure. In Experiment 2 on another set of subjects, the IOP was increased by 30 mmHg from the baseline for 10 minutes. The LSFG images were recorded before, immediately after the IOP elevation, time 0, and at 1 (time 1), 3 (time 3), 5 (time 5), 7 (time 7), and 10 (time 10) minutes while the IOP was elevated. The LSFG images were also recorded at 1 (time 11), 3 (time 13), and 5 (time 15) minutes after the release of the pressure on the eye.

### Laser speckle flowgraphy (LSFG)

The LSFG-NAVI (Softcare Co., Ltd., Fukutsu, Japan) instrument was used to measure the blood flow on the ONH. The principles of LSFG have been described in detail^45–48^. Briefly, this instrument consists of a fundus camera equipped with an 830 nm diode laser as the light source and a standard charge-coupled device sensor as the detector. After switching on the laser, a speckle pattern of the fundus appears due to the interference of the light scattered by the movements of the erythrocytes in the retinal vessels. The MBR is a measure of the relative blood flow velocity, and it is determined by examining the pattern of the speckle contrast produced by the movements of the erythrocytes in the ocular blood vessels.

To evaluate the circulation on the ONH, a circular marker was set surrounding the ONH (Fig. 1B). The “vessel extraction” function of the software then identified the vessel and tissue areas on the ONH so that the MBR of each area could be assessed separately (Fig. 1C). The MBR of the vessel (MBR-vessel) and tissue areas (MBR-tissue) on the ONH were determined. The LSFG was measured two times at each time point in all eyes. The average of the MBRs was calculated and used for the statistical analyses.

### Statistical analyses

The values are presented as the means ± standard deviations. Independent *t* tests were used to determine the significance of the differences in the normally distributed values. Spearman analyses were used to determine the coefficients of correlation between the variables. A mixed linear model was used to incorporate the appropriate covariates between the repeated measured values over time. All statistical analyses were performed using IBM SPSS Statistics for Windows, Version 23 (IBM Corp., Armonk, NY). The significance level was set at a probability (*P*) value of < 0.05.

## Electronic supplementary material

supplemental video
